# CircPTPRA acts as a tumor suppressor in bladder cancer by sponging miR-636 and upregulating KLF9

**DOI:** 10.18632/aging.102530

**Published:** 2019-12-10

**Authors:** Qingqing He, Lifang Huang, Dong Yan, Junming Bi, Meihua Yang, Jian Huang, Tianxin Lin

**Affiliations:** 1 Department of Urology, Sun Yat-sen Memorial Hospital, Sun Yat-sen University, Guangzhou, China; 2 Guangdong Provincial Key Laboratory of Malignant Tumor Epigenetics and Gene Regulation, Sun Yat-sen Memorial Hospital, Sun Yat-sen University, Guangzhou, China

**Keywords:** bladder cancer, circPTPRA, miR-636, KLF9, proliferation

## Abstract

Growing evidence suggests that circular RNAs (circRNAs) play pivotal roles in cancer progression. In this study, bioinformatic analysis identified a dysregulated circRNA termed circPTPRA in bladder cancer (BC). By using qRT-PCR analysis, we verified that circPTPRA is down-regulated in clinical BC specimens compared with the matched non-tumor samples, while correlation analyses showed that low circPTPRA expression is associated with poor prognosis, advanced tumor stage and larger tumor size. Based on these findings, we conducted functional assays and revealed that circPTPRA inhibits BC cell proliferation in vitro and tumor growth in vivo. In addition, RNA pull-down, miRNA capture, FISH, and luciferase reporter assays demonstrated that circPTPRA can directly sponge miR-636. Cell transfection experiments showed that miR-636 promotes the proliferation of BC cells by decreasing the expression of Krüppel Like Factor 9 (KLF9) upon binding to the 3’UTR of its mRNA.

Further analysis confirmed that circPTPRA competitively sponges miR-636 to upregulate the KLF9 expression, leading to decreased proliferation of BC cells. Our investigation indicates that circPTPRA acts as a tumor suppressor in BC, and suggests that this circRNA may be a novel prognostic biomarker and therapeutic target in BC.

## INTRODUCTION

Bladder cancer is the ninth most common cancer worldwide with an estimated 549, 000 newly diagnosed cases and 200, 000 deaths in 2018 [1, 2]. Most BC cases are diagnosed as non-muscle invasive BC (NMIBC), which has favorable prognosis following transurethral resection and intravesical perfusion [3]. Unfortunately, NMIBC always relapses and progresses to muscle invasive BC (MIBC), which often metastasizes to lymph node and distant organs [4]. Despite standard treatment strategies such as surgery, radiotherapy and chemotherapy, BC metastasis is seldom controlled and leads to poor prognosis. The proliferation of BC and its infiltration depth promote metastasis [5]. Thus, elucidating the mechanisms underlying BC cell proliferation is crucial to design new approaches to prevent metastasis.

Circular RNAs (circRNAs) constitute a large class of single-stranded, non-coding RNA molecules that attracted great research interest in recent years. CircRNAs are generated by back-splicing of pre-mRNA and form covalently closed, continuous loop structures without terminal 5’ caps and 3’ polyadenylated tails [6, 7]. Initially, circRNAs were misinterpreted as the byproducts of splicing errors [8]. With the development of high-throughput sequencing and bioinformatic analysis, many circRNAs have been discovered in multiple species; based on functional assays, regulatory roles for circRNAs in biological processes, including cancer, have been proposed [9–11]. These include translation modulation by microRNA (miRNA) sponging, transcriptional regulation, protein binding, and encoding protein [12–15].

The most studied circRNA role is arguably miRNA sponging. By inhibiting the association of miRNAs with the UTRs of mRNAs, circRNAs are considered competitive endogenous RNAs (ceRNAs) that modulate protein translation [12]. Emerging evidence revealed that several circRNAs may influence tumor proliferation, invasion and tumor immunity, and may aid prognosis assessment in several cancers [16–18]. For example, circPVT1 promotes gastric cancer proliferation by sponging members of the miR-125 family and was proposed as an independent prognostic marker for overall survival [16]. CircABCB10 modulates proliferation and progression of breast cancer by sponging miR-1271 [18], and circTP63 promotes lung squamous cell carcinoma progression by competitively binding to miR-873-3p and upregulating FOXM1 [19]. Although several studies assessed the roles of circRNAs in BC progression, the mechanisms by which they affect proliferation of BC cells remain largely unknown [12, 20].

Here, we analyzed the circRNA profile of three clinical BC samples and paired adjacent normal bladder tissues retrieved from the GEO database (GEO97239), and identified circPTPRA (circBase ID: hsa_circ_0006117) as a dysregulated circRNA in BC. Subsequently, the correlation between circPTPRA expression and clinical parameters, and functional assays were conducted to investigate potential functions of circPTPRA in BC and identify miRNA and gene targets. Our results indicate circPTPRA acts as a tumor suppressor in BC, and suggest a potential role for this novel circRNA as a prognostic biomarker and therapeutic target.

## RESULTS

### Identification and characterization of circPTPRA

**Figure 1:**
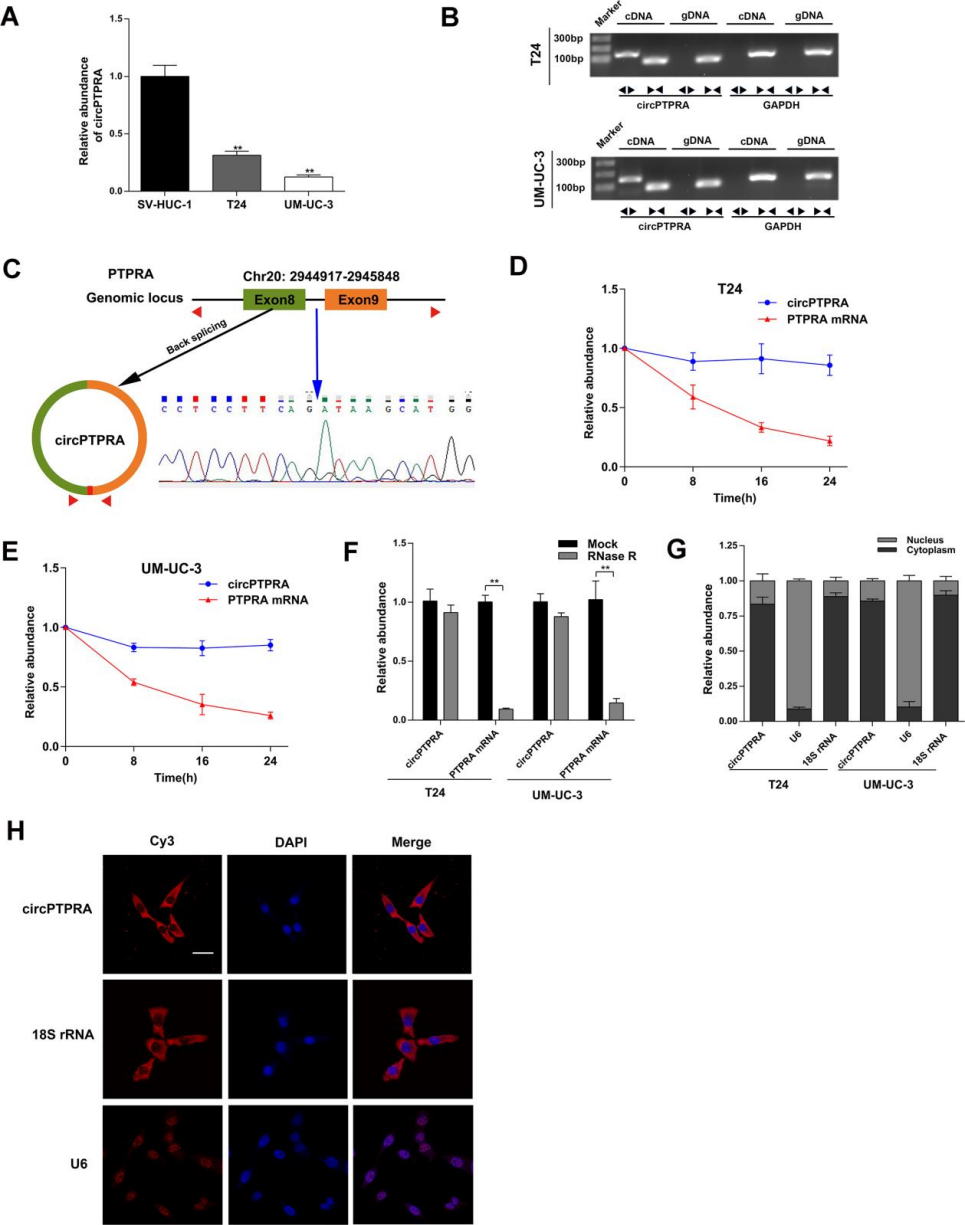
**Characterization of circPTPRA in BC cell lines.** (**A**) Expression of circPTPRA in normal SV-HUC-1 cells and two BC cell lines (T24 and UM-UC-3). (**B**) Gel electrophoresis of qRT-PCR products resulting from divergent and convergent primers. GAPDH was used as internal control. (**C**) Schematic diagram depicting the circPTPRA’s origin from exons 8 and 9 of the *PTPRA* gene. Sanger sequencing confirmed the back-splicing junction site (blue arrow). (**D**, **E**) Analysis of PTPRA mRNA and circPTPRA by qRT-PCR in BC cell lines after actinomycin D treatment. (**F**) PTPRA mRNA and circPTPRA levels measured by qRT-PCR after RNase R treatment in BC cell lines. (**G**) Cellular localization of circPTPRA in BC cell lines, as assessed by cytoplasmic and nuclear fractionation assay. (**H**) FISH assay of indicating the cellular distribution of circPTPRA in UM-UC-3 cells. Scale bar=50μm. Data are presented as mean ± SD. ^*^P < 0.05, ^**^P < 0.01 (Student’s t-test).

To identify dysregulated circRNAs in BC, we screened three matched BC and adjacent normal bladder tissue sets retrieved from the GEO database (GEO97239). We found that circPTPRA (circBase ID: hsa_circ_0006117) was significantly downregulated in BC samples, and selected this circRNA for further characterization. Expression analysis using qRT-PCR indicated lower circPTPRA levels in two BC cell lines, T24 and UM-UC-3, compared to normal bladder epithelial SV-HUC-1 cells (Figure 1A). Because circPTPRA originates from PTPRA (protein tyrosine phosphatase receptor type A) mRNA, the existence of circPTPRA was verified to exclude the possibility that it resulted from a gene rearrangement event. Thus, we designed divergent primers for circPTPRA and convergent primers for PTPRA. Agarose gel electrophoresis indicated that circPTPRA was only amplified by divergent primers using cDNA but not gDNA from BC cells (Figure 1B). By browsing the human reference genome (GRCh37/hg19), we identified that circPTPRA was derived from the exons 8 and 9 of the *PTPRA* gene (Figure 1C). Sanger sequencing of PCR products of divergent primers validated the existence of the back-splicing junction site of circPTPRA (Figure 1C). Additionally, an actinomycin D assay revealed that circPTPRA was more stable than the linear PTPRA mRNA, and its half-life was more than 24h (Figure 1D, 1E). Moreover, an RNase R assay showed that circPTPRA was resistant to RNase R, whereas PTPRA mRNA was not (Figure 1F). To identify the location of circPTPRA in BC cells, we conducted a nuclear and cytoplasmic extraction assay which indicated that circPTPRA was mostly located in the cytoplasm of BC cells (Figure 1G). The same result was obtained through FISH assay (Figure 1H).

### Expression of circPTPRA in human BC specimens and clinical significance

**Figure 2:**
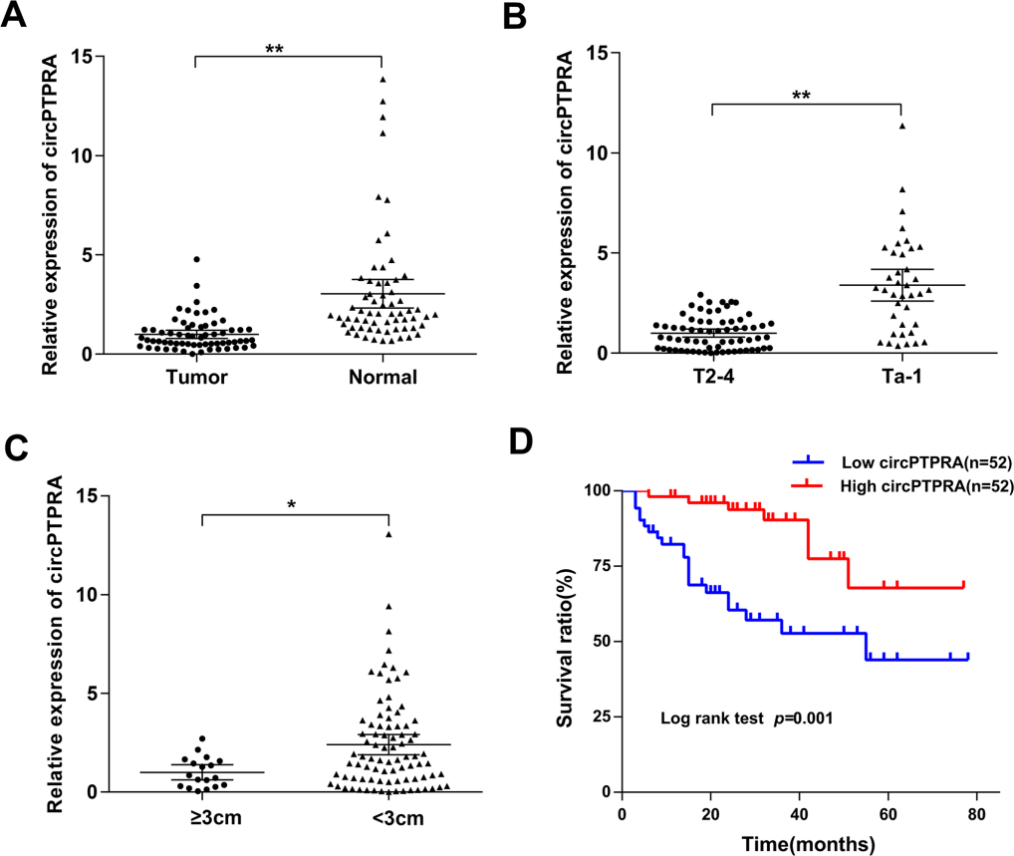
**Expression of circPTPRA in human BC specimens.** (**A**) Relative expression of circPTPRA in BC samples and matched adjacent normal tissues (Wilcoxon matched-pairs signed rank test). (**B**) Relative expression of circPTPRA according to BC clinical T stage (Mann-Whitney U test). (**C**) Expression of circPTPRAaccording to BC clinical tumor size (Mann-Whitney U test). (**D**) Kaplan-Meier analysis of overall survival in BC patients. Data are presented as the mean and 95% CI. ^*^P< 0.05, ^**^P < 0.01

To further verify the expression of circPTPRA in BC, 64 matched BC and adjacent normal specimens were analyzed by qRT-PCR. Results confirmed that circPTPRA was downregulated in BC tissues compared with normal tissues (Figure 2A). Additionally, we analyzed the expression of circPTPRA in 104 BC specimens and found that both advanced tumor stage (T2-T4) and tumor size (≥3cm) correlated with low circPTPRA expression (Figure 2B, 2C). Then we divided patient samples into high and low circPTPRA groups, and the Chi-square test indicated that circPTPRA expression was indeed associated with tumor stage and size, but not with other clinical parameters (Table 1). Moreover, survival analyses indicated poor prognosis for BC patients with low circPTPRA expression (Figure 2D).

**Table 1:** Correlation between circPTPRA expression and clinicopathological characteristics of bladder cancer patients.

**Variable**		**Cases**	**CircPTPRA**		***P***
			**Low**	**High**	
Age(y)					
	<65	55	28	27	0.844
	≥65	49	24	25	
Gender					
	Male	91	44	47	0.374
	Female	13	8	5	
Tumor stage					
	Ta-T1	38	9	29	**0.000**
	T2-T4	66	43	23	
LN status					
	LN-	90	42	48	0.149
	LN+	14	10	4	
Multifocality					
	Unifocal	92	45	47	0.539
	Multifocal	12	7	5	
Histological grade					
	Low	12	4	8	0.358
	High	92	48	44	
Tumor size					
	<3cm	86	39	47	**0.038**
	≥3cm	18	13	5	
Total		104	52	52	

^*^P<0.05 was considered statistically significant (Chi-square test).

### circPTPRA inhibits the proliferation of BC cells in vitro

**Figure 3:**
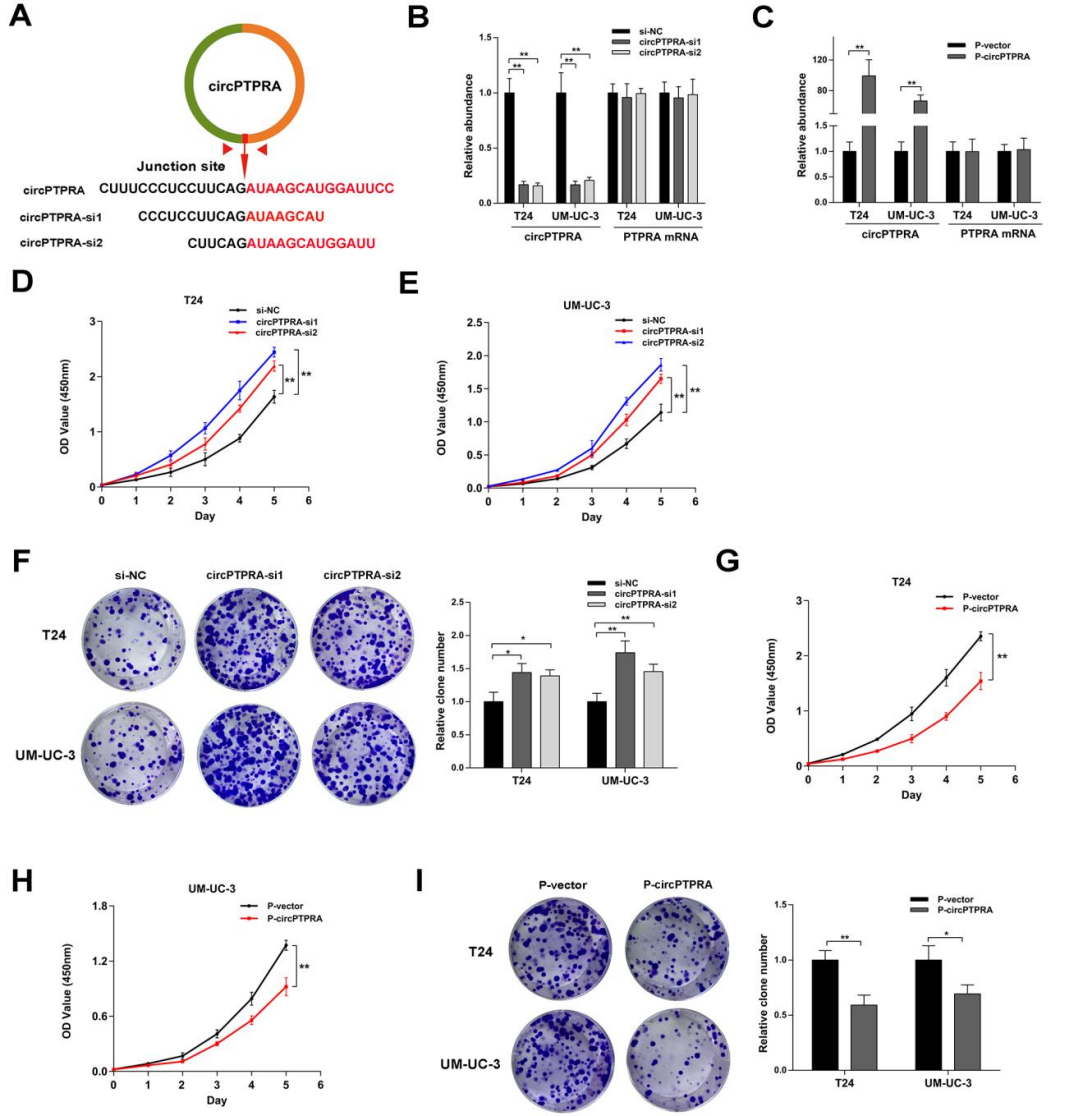
**CircPTPRA inhibits the proliferation of BC cells.** (**A**) Design of siRNAs targeting circPTPRA. (**B**, **C**) Expression of circPTPRA in BC cell lines after siRNAs treatment and lentivirus transfection. (**D**–**F**) Effects of circPTPRA silencing on BC cell proliferation measured through CCK-8 and colony formation assays. (**G**–**I**) Effects of circPTPRA overexpression on BC cell proliferation detected by CCK-8 and colony formation assays. Data are presented as the mean ± SD of three experiments. ^*^P< 0.05, ^**^P< 0.01(Student’s t-test).

To illustrate the function of circPTPRA in BC cells, gain- and loss-of-function assays were performed in vitro. First, two siRNAs targeting the back-splicing junction site of circPTPRA were designed (Figure 3A). The expression of circPTPRA was significantly decreased after transfection of these siRNAs in BC cells, but without obvious changing in the level of PTPRA mRNA (Figure 3B). After construction of BC cell lines with stable expression of circPTPRA, qRT- PCR analysis confirmed that circPTPRA was overexpressed in those cells, but not in cells expressing a control vector (Figure 3C). Next, cell viability and colony formation assays showed that circPTPRA knockdown promoted the proliferation of BC cells (Figure 3D–3F). Conversely, and as expected, overexpression of circPTPRA inhibited BC cells proliferation (Figure 3G–3I).

### circPTPRA acts as a sponge of miR-636

**Figure 4:**
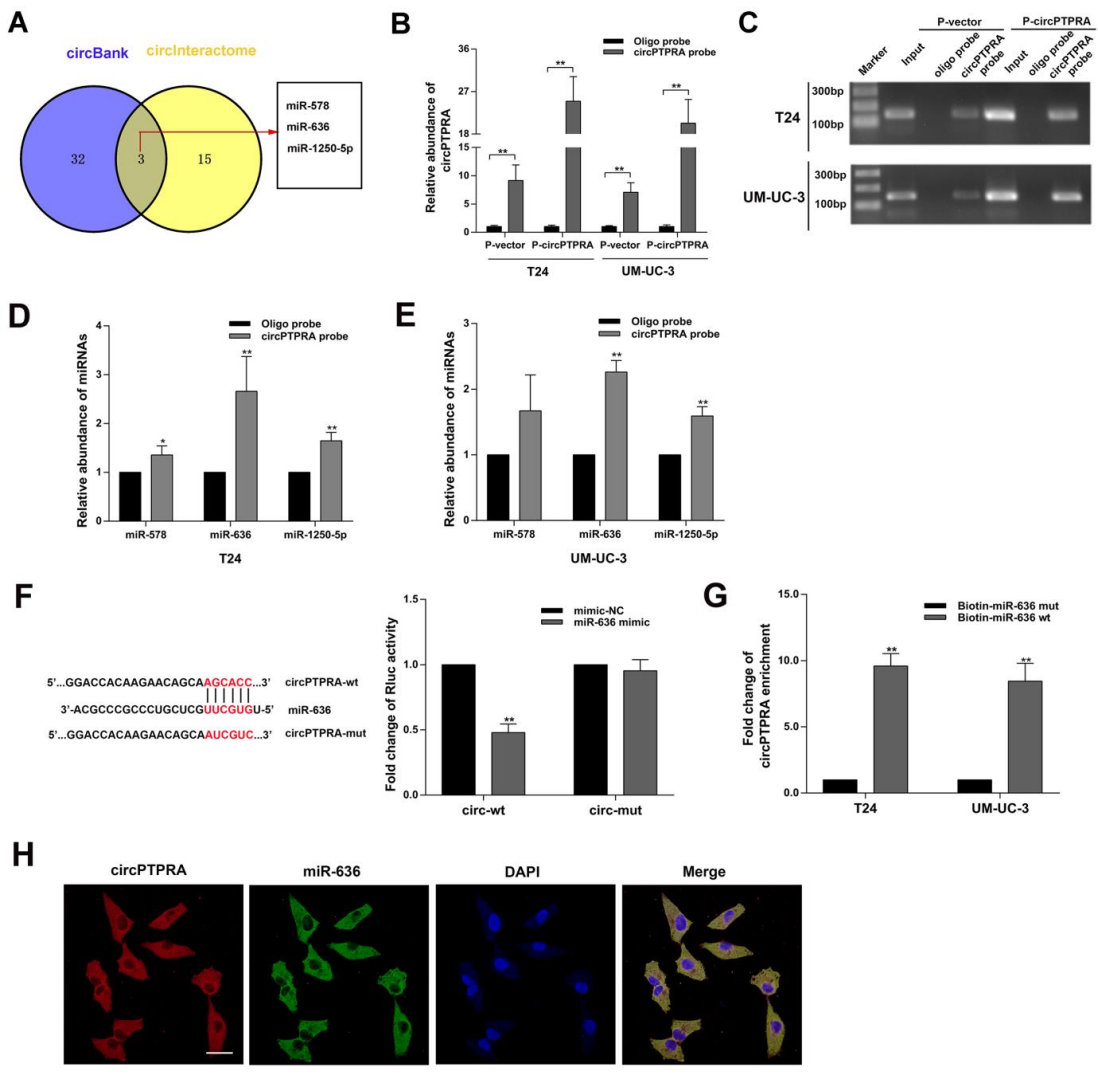
**CircPTPRA acts as a sponge of miR-636.** (**A**) Predicted miRNA target of circPTPRA identified on circInteractome and circBank. (**B**, **C**) Expression of circPTPRA detected by qRT-PCR analysis and gel electrophoresis of samples obtained by RNA pull-down assay. (**D**, **E**) Analysis of miRNAs bound to circPTPRA and control probes, measured by qRT-PCR. (**F**) Luciferase reporter assay results demonstrating the interaction between circPTPRA and miR-636. (**G**) Enrichment of circPTPRA captured by biotin-coupled miR-636 mimic or its mutant variant, as detected by qRT-PCR. (**H**) Co-localization of circPTPRA and miR-636 determined by FISH assay. Scale bar=50μm. Data are presented as the mean ± SD of three experiments. ^*^P < 0.05, ^**^P < 0.01(Student’s t-test).

The cytoplasmic localization of circPTPRA in BC cells implied that it might work by sponging miRNAs. Thus, we searched the circBank (http://www.circbank.cn/searchMiRNA.html) and circInteractome (https://circinteractome.nia.nih.gov/index.html) databases to predict potential miRNAs targets of circPTPRA. Three candidate miRNAs overlapping between the two databases were retrieved (Figure 4A).

**Figure 5:**
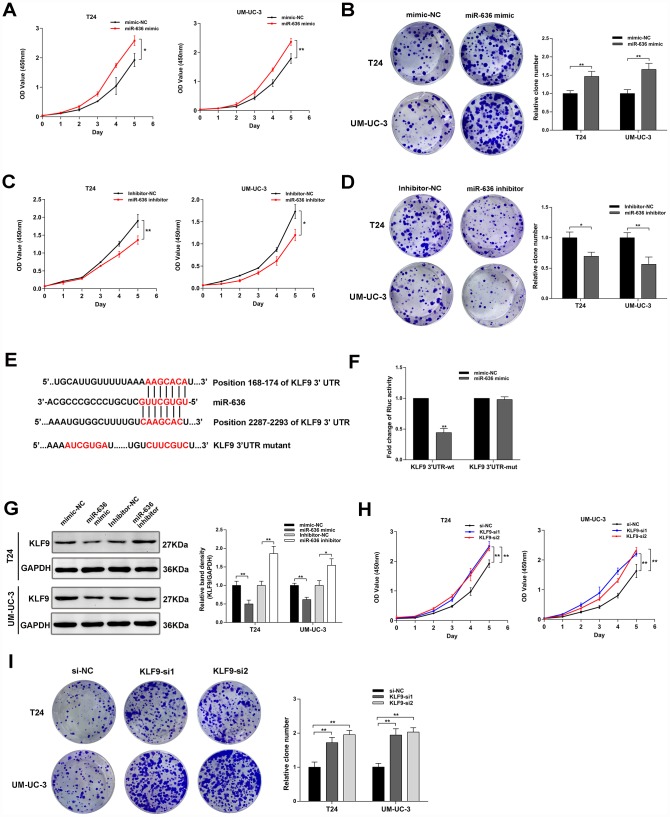
**MiR-636 promotes proliferation in BC cell lines.** (**A**–**D**) Results of cell proliferation and colony formation assays in BC cell lines transfected with miR-636 mimic and miR-636 inhibitor. (**E**) Schematic diagram depicting the interaction of miR-636 and the 3’UTR of KLF9 mRNA. (**F**) Luciferase reporter assay results in BC cells co-transfected with miR-636 and KLF9 3’UTR. (**G**) Expression of KLF9 detected by western blot in BC cells transfected with miR-636 mimic and miR-636 inhibitor. (**H**, **I**) Results of CCK-8 and colony formation assays on BC cell lines transfected with KLF9 siRNAs. Data are presented as the mean ± SD of three experiments. ^*^P < 0.05, ^**^P < 0.01(Student’s t-test).

After performing RNA pull-down assay, the capture specificity of the biotin-coupled circPTPRA probe was validated by qRT-PCR and gel electrophoresis (Figure 4B, 4C). Moreover, following qRT-PCR analysis of RNAs bound to the circPTPRA probe-coated beads, abundant enrichment for miR-636 was detected (Figure 4D, 4E). Subsequently, luciferase reporter assays indicated that miR-636 decreased the Rluc activity of the circPTPRA psiCHECK-2 plasmid but had no effect on circPTPRA psiCHECK-2 mutant type (Figure 4F). Furthermore, a biotin-coupled miR-636 mimic capture assay also showed that circPTPRA was enriched by miR-636. While, mutating the circPTPRA binding site in miR-636 abolished this effect (Figure 4G). On the other hand, the co-localization of circPTPRA and miR-636 in the cytoplasm of UM-UC-3 cells (Figure 4H). Taken together, these results validated the association between circPTPRA and miR-636. To further assess the biological effects of the miR-636, we conducted cell viability and colony formation assays. Results revealed that miR-636 mimic promoted the proliferation of BC cells (Figure 5A, 5B), while miR-636 inhibitor had the opposite effect (Figure 5C, 5D).

### KLF9 acts as the target gene of miR-636 and inhibits BC cells proliferation

Based on results from Targetscan (http://www.targetscan.org/vert_72/) and miRDB (http://mirdb.org/) databases, we focused on the *KLF9* gene, whose transcript contains the binding site for miR-636 in its 3’UTR. After constructing a luciferase reporter plasmid containing wild-type KLF9 3’UTR or a mutant type KLF9 3’UTR variant (Figure 5E), reporter assay results demonstrated that miR-636 significantly decreased the Rluc activity of KLF9 3’UTR, but did not affect the activity of its mutant form (Figure 5F). In turn, western blot analysis showed that miR-636 negatively regulated the expression of *KLF9* (Figure 5G), while in vitro assays indicated that *KLF9* silencing promoted the proliferation of BC cell lines (Figure 5H, 5I)

### circPTPRA regulates the expression of KLF9 by sponging miR-636

**Figure 6:**
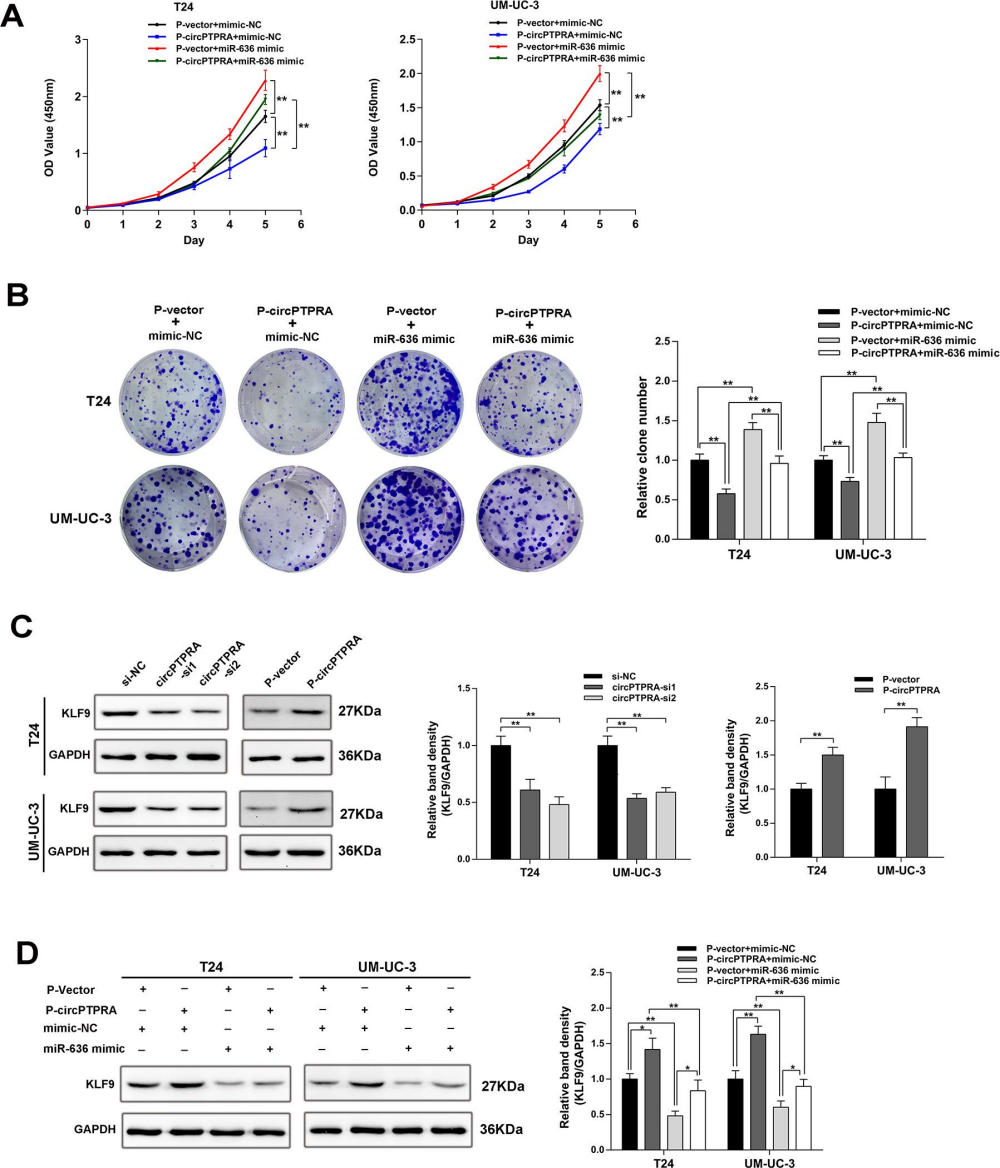
**CircPTPRA regulates the expression of KLF9 by sponging miR-636.** (**A**, **B**) The proliferative effect of circPTPRAon BC cells was partially abrogated by miR-636, evidenced by CCK-8 and colony formation assays. (**C**) Western blot results showing that circPTPRA upregulates the expression of KLF9. (**D**) Western blot results demonstrating that the upregulation of KLF9 by circPTPRA is partially abrogated by miR-636. Data are presented as the mean ± SD of three experiments. ^*^P< 0.05, ^**^P< 0.01(Student’s t-test).

Given that circPTPRA and KLF9 mRNA shared a common miR-636 binding motif, we inferred that circPTPRA might regulate the expression of *KLF9* by competitively sponging miR-636. A functional assay revealed that miR-636 mimic partially reversed the inhibition of cell proliferation induced by circPTPRA (Figure 6A, 6B). In addition, western blot showed a positive association between *KLF9* and circPTPRA expression (Figure 6C). After co-transfection of circPTPRA and miR-636 mimic, partial abrogation of the repression exerted by miR-636 on *KLF9* was evident (Figure 6D). Taken together, these data suggest that circPTPRA restricts the proliferation of BC cells by suppressing miR-636-mediated silencing of KLF9 mRNA translation.

### circPTPRA represses tumor growth in vivo

**Figure 7:**
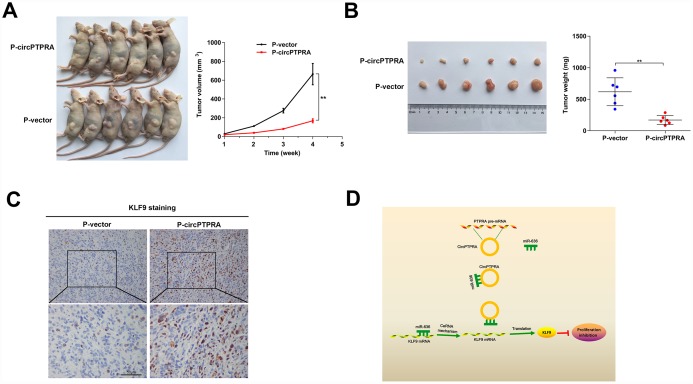
**CircPTPRA overexpression inhibits BC growth in vivo.** (**A**)Tumor growth measurements in mice injected with circPTPRA-overexpressing cells or vector control cells. (**B**) Representative image of subcutaneous tumors generated in BALB/c nude mice (left), and corresponding tumor weights (right). (**C**) IHC staining of KLF9 in excised tumors. (**D**) Schematic diagram depicting the inhibitory effect of circPTPRA on BC cell proliferation by impeding the miR-636/KLF9 interaction. Data are presented as the mean ± SD. ^**^P< 0.01(Student’s t-test).

To evaluate the effect of circPTPRA on BC growth in vivo, we stably expressed circPTPRA in UM-UC-3 cells, and injected them subcutaneously to generate tumor xenografts in nude mice. After 4 weeks, delayed the tumor growth was observed in mice injected with circPTPRA-transfected UM-UC-3 cells, compared with animals in the vector control group (Figure 7A). Consistently, the weight of circPTPRA-overexpressing tumors was lower than that of controls (Figure 7B).

Moreover, IHC analysis indicated that overexpression of circPTPRA was correlated with upregulation of KLF9 in tumor samples (Figure 7C).

## DISCUSSION

Despite clinical treatments such as surgery and chemotherapy, advanced BC characterized by muscle infiltration and metastasis is still associated with poor prognosis and elevated mortality [1, 4, 5]. Although several circRNAs have emerged as potential prognostic biomarkers or therapeutic targets in BC, the molecular mechanisms by which they influence proliferation and survival of BC cells remain largely unknown [20, 21]. Expression profiling is an effective method to identify novel tumor suppressor or oncogenic circRNAs. Using this approach, we found that circPTPRA was dysregulated in clinical BC specimens retrieved from a GEO database. Further analysis showed that low circPTPRA expression in samples from BC patients was associated with poor prognosis, advanced tumor stage, and increased tumor size. Meanwhile, functional assays indicated that circPTPRA inhibited the proliferation of BC cells by inhibiting the miR-636/KLF9 interaction.

CircRNAs possess miRNA response elements (MRE) through which they bind to miRNAs and prevent them from binding their target mRNAs [9]. The function of circRNAs as miRNAs sponges is most frequently explored in tumors [22]. For example, the prominent circRNA ciRS-7 has multiple binding sites for miR-7, which was shown to promote or restrain tumor progression depending on tumor type by modulating the expression of several oncogenes [23]. Another circRNA, hsa_circ_0020123 promotes oncogenesis by upregulating ZEB1 and EZH2 upon competitive binding and suppression of miR-144 [24]. Likewise, hsa-circ-001569 may promote the proliferation and invasion of colorectal cancer cells by sponging miR-145 and subsequently upregulating the expression of E2F5, BAG4, and FMNL2 [25]. Several circRNAs have been proposed to influence BC biology. We recently showed that circBPTF promotes BC progression by disrupting the miR-31-5p/RAB27A interaction [26]. Li et al. reported that circHIPK3 functions as a tumor suppressor in BC, attenuating the migration, invasion, and angiogenesis by sponging miR-558 [12]. On the other hand, Zheng et al. showed that overexpression of circNR3C1 significantly inhibits the proliferation of BC cells by sponging miR-27a-3p and downregulating cyclin D1 expression [27]. Here, we revealed that circPTPRA is downregulated in human BC specimens, whereas forced expression inhibited the proliferation of BC cells in vitro and attenuated tumorgenesis in vivo. Since the location of circPTPRA in BC cells was mainly cytoplasmatic, we hypothesized that circPTPRA modulated proliferation by sponging miRNAs.

Bioinformatic analyses predicted several miRNAs with binding sites for circPTPRA, and our RNA pull-down assays demonstrated that circPTPRA captured abundant levels of miR-636. We also found that miR-636 reduced the Rluc activity of circPTPRA on a luciferase reporter assay by at least 50%. As expected, a functional assay revealed that miR-636 effectively competed with circPTPRA. Among potential targets of miR-636, the *KLF9* gene emerged as a candidate on both TargetScan and miRDB databases. Accordingly, luciferase reporter assays indicated that miR-636 decreased the Rluc activity of KLF9 3’UTR embedded in the psiCHECK-2 plasmid, while western blot demonstrated that miR-636 negatively regulated KLF9 protein levels. Evidence for the involvement of *KLF9* in cancer was provided by previous studies. For example, the expression of *KLF9* was shown to be inversely correlated with endometrial tumor grade [28], and its silencing was further proposed to contribute to the etiology of endometrial cancer initiated by aberrant ESR1 signaling [29]. Additional evidence supporting a role for *KLF9* as a tumor suppressor from another report indicated that KLF9 inhibits the growth of hepatocellular carcinoma cells by modulating p53 [30].

Since miR-636 shares a common binding motif with circPTPRA and KLF9 mRNA, we hypothesized that circPTPRA might modulate the expression of *KLF9* by competitively binding miR-636. Indeed, western blot analysis confirmed that circPTPRA positively regulated KLF9 levels, and partially reversed the inhibitory effect of miR-636 mimic. Moreover, stimulatory effect on BC cell proliferation mediated by miR-636 mimic was also abrogated by overexpression of circPTPRA. Thus, we suggest that circPTPRA exerts a tumor suppressor function in BC cells by preventing the miR-636/KLF9 association.

In summary, our present work identified a novel circRNA termed circPTPRA, which is downregulated in clinical BC specimens. Furthermore, we showed that low circPTPRA expression in BC is associated with poor prognosis, advanced tumor stage, and increased tumor size. Given its tumor-suppressor role as inhibitor of the miR-636/KLF9 interaction, circPTPRA emerges as a potential novel prognostic biomarker and therapeutic target for BC.

## MATERIALS AND METHODS

### Ethic statement and clinical samples

A total of 104 BC specimens and 64 paired, adjacent normal bladder tissue samples were obtained from patients who underwent surgery in Sun Yat-sen Memorial Hospital, Sun Yat-sen University between 2011 and 2017. This study was approved by the Ethics Committee of Sun Yat-sen Memorial Hospital, and written informed consent was obtained from all participants. Histological and pathological diagnoses of BC were confirmed by two experienced pathologists according to the 7^th^ edition of the TNM classification of the International Union Against Cancer (UICC, 2009).

### Cell culture

Two BC cell lines (T24 and UM-UC-3), the immortalized human normal bladder epithelial cell line SV-HUC-1, and the human embryonic kidney cell line HEK-293T were purchased from American Type Culture Collection. Culture media (Gibco, USA) included DMEM (UM-UC-3 and HEK-293T cells), RPMI 1640 (T24 cells), and Ham’s F-12K (SV-HUC-1 cells). The culture medium of cell lines was supplemented with 10% fetal bovine serum (BI, Israel) and 1% penicillin/streptomycin (Gibco, USA). All cells were incubated in a humidified atmosphere with 5% CO_2_ at 37°C.

### RNA isolation and quantitative real-time reverse transcription PCR (qRT-PCR)

RNAiso Plus (TaKaRa, Japan) was applied to extract total RNA from tissues or cells. Nuclear and Cytoplasmic Extraction Kit (Life Technologies, USA) was used to isolate RNA from nucleus and cytoplasm following manufacturer’s instructions. The Genomic DNA from BC cell lines was isolated using the MiniBEST Universal Genomic DNA Extraction Kit Ver.5.0 (Takara, Japan). The Complementary DNA was synthesized using the Prime Script RT Master Mix kit (Takara, Japan). TB Green Premix Ex Taq II (Takara, Japan) and a Quantstudio™ DX system (Applied Biosystems, Singapore) were used for qRT-PCR assays. Levels of circRNA and mRNA were normalized to GAPDH, and miRNA levels were normalized to small nuclear U6 RNA. Relative expression level was calculated with the 2^−ΔΔCt^ method.

### Actinomycin D assay and RNase R treatment

T24 and UM-UC-3 cell cultures were supplemented with 2μg/mL actinomycin D (Sigma, USA) for various time points. Total RNA extracted from T24 and UM-UC-3 cells was treated with RNase R (Epicenter Technologies, USA) according to manufacturer’s instructions for 30min at 37 °C. The stability of circPTPRA and PTPRA mRNA was analyzed by qRT-PCR.

### Fluorescence in situ hybridization (FISH)

BC cells were cultured in confocal dishes to 50% confluence. After washing three times with PBS, the cells were fixed in 4% paraformaldehyde for 15 min. FISH staining was carried out using a FISH Kit (GenePharma, China). To identify circPTPRA, we used FISH probes (GenePharma, China) hybridizing with back-splicing junction site of circPTPRA. Nuclear staining was performed with 4, 6-diamidino- 2- phenylindole (DAPI). FISH images were captured with a Zeiss LSM800 confocal microscope (Carl Zeiss AG, Germany).

### Cell transfection and vector construction

Lipofectamine RNAiMax (Invitrogen, USA) was used to transfect siRNA and miRNA mimics and inhibitors (GenePharma, Shanghai, China) into cells. To construct the overexpression plasmid, the sequence of circPTPRA was cloned into a plenti-ciR-GFP -T2A vector (IGE Biotech Co, China). The corresponding sequences were inserted into a psiCHECK-2 vector (Synbio Tech, China) to synthesize luciferase reporter plasmids. Plasmid transfection was conducted using X-tremeGENE (Sigma, USA). Generation of stably transfected cell lines and lentivirus packaging were carried out as previously described [21].

### Cell viability assay

Transfected cells suspended in 100μl complete culture medium were seeded into 96-well plates at a density of 1,000 cells per well for 6 days. Cell viability was measured using the Cell Counting Kit-8 (CCK-8; Dojindo Laboratories, Japan) according to manufacturer’s instructions. Briefly, 10μl CCK-8 reagent was added into each well, and 2 h later absorbance was recorded at 450 nm on a Spark 10M microplate reader (Tecan, Austria).

### Colony formation assay

Transfected cells were seeded in 6-well plates at a density of 1000 cells/well, and the culture medium was changed every 3 days. After 10 to 14 days, the cells were washed with PBS, fixed in 4% paraformaldehyde for 30 min, and stained with 0.2% crystal violet for 30 min. Cell colonies were photographed and counted. Three independent experiments were performed.

### RNA pull-down assay

The RNA pull-down assay was performed as previously described [21]. Briefly, streptavidin-coupled magnetic beads (Life Technologies, USA) were incubated with biotin-coupled circPTPRA probes and random oligo probes for 2 h at room temperature to generate probe-coated beads. Approximately 1× 10^7^ BC cells were collected, lysed, and sonicated, and cell lysates were incubated with probe-coated beads at 4°C overnight. Bead-bound RNAs were washed in washing buffer and extracted by RNAiso Plus (TaKaRa, Japan) for qRT-PCR analysis.

### Biotin-coupled miRNA capture assay

Biotin-coupled miR-636 mimic (or its mutant variant) was transfected into BC cell lines stably expressing circPTPRA using Lipofectamine RNAiMax. After 48 h, the cells were harvested and lysed. To generate blocked beads, streptavidin-coupled magnetic beads were washed with lysis buffer and blocked with yeast tRNA on a rotator at 4 °C for 2 h. The cell lysates were incubated with blocked beads at 4°C overnight. After washing the beads, bound RNAs were purified using an RNeasy Mini Kit (QIAGEN, Germany) and analyzed by qRT-PCR.

### Luciferase reporter assay

The sequences of circPTPRA and its mutant form were cloned into a psiCHECK-2 vector. Luciferase reporter plasmids containing either the KLF9 wild-type 3’UTR or its mutant type were also constructed. After cotransfection of miR-636 mimic and the circPTPRA luciferase reporter plasmid (or its mutant variant) into HEK-293T cells for 48h, luciferase activity was measured by a dual-luciferase reporter assay system (Promega, Madison, WI, USA) according to the manufacturer’s protocol. Furthermore, luciferase activity was also detected after cotransfection of KLF9 3’UTR (or its mutant form) and miR-636 mimic. Relative Renilla luciferase (Rluc) activity was normalized to firefly luciferase activity. This assay was performed three times independently.

### Xenograft model

Male BALB/c nude mice (4 weeks old) were purchased from the Experimental Animal Center, Sun Yat-sen University. Animal care was conducted following the Guide for the Care and Use of Laboratory Animals (NRC, USA), and all animal experiments were approved by the Ethics Committee of Sun Yat-sen University. The mice were randomly divided into two groups (n=6 per group). UM-UC-3 cells (5×10^6^) stably expressing circPTPRA or control vector were resuspended in 100μl PBS and injected subcutaneously into the left side of the dorsum. Tumor size was measured with a caliper every week, and volume was calculated with the formula length × width^2^×0.5. After four weeks, the mice were sacrificed and tumors were weighed and processed for further experiments.

### Western blot

Proteins were extracted using radio immunoprecipitation assay (RIPA) lysis buffer (Beyotime, China), and concentrations determined with a BCA Kit (Thermo, USA). Protein samples (35μg) were separated by 10% SDS-PAGE and transferred onto PVDF membranes (Millipore, USA). After blocking in 5% non-fat milk for 1h, the membranes were incubated with anti-KLF9 (1:1,000; Abcam, ab170980) and anti-GAPDH (1:10000; Abcam, ab181602) antibodies at 4°C overnight. HRP-conjugated secondary antibody was next applied for 1 h at room temperature. Signals were visualized with an ECL Kit (Millipore, Germany) and captured using an iBrightCL1000 imaging system (Thermo, USA). The optical density of the protein bands was quantified by ImageJ software 1.48 (National Institutes of Health, Bethesda, MD, USA).

### Immunohistochemistry (IHC)

Paraffin-embedded tissues were cut into 4 μm slides and IHC was performed as previously described [31] using a primary antibody directed against KLF9(1:400; Abcam, ab170980).

### Sequences information

The sequences of the primers, oligonucleotides, and probes used in this study are provided in the Supplementary Table 1.

### Statistical analysis

Statistical analyses were performed using SPSS 19.0 statistical software. All data are presented as mean ± standard deviation (SD) unless specifically stated. Comparison between two groups was performed by student’s t test, Wilcoxon rank-sum test, or Mann-Whitney U test. The association between circPTPRA expression levels and clinical parameters was evaluated by Chi-square test. Kaplan-Meier curves and log-rank test were applied for overall survival analysis. *P* < 0.05 was considered significant.

## Supplementary Material

Supplementary Table 1
