# Supplementary material for: CircPTPRA acts as a tumor suppressor in bladder cancer by sponging miR-636 and upregulating KLF9
Source: Aging (Albany NY). 2019 Dec 10;11(23):11314–28. doi: 10.18632/aging.102530 (PMC6932899; doi:10.18632/aging.102530)
Supplement: Supplementary Table 1 [file aging-11-102530-s001..pdf]

SUPPLEMENTARY TABLE

Supplementary Table 1. The sequences of primers, oligonucleotides and probes used in this study.

**Primers**	
CircPTPRA Forward	TAACCAGTTCACGGATGCCA
CircPTPRA Reverse	AGCATTGTTGGCACTGACAC
Linear PTPRA Forward	ACCAGGGAGAATAAGAGCCG
Linear PTPRA Reverse	GATCATGCCCTTTCCGTCAC
GAPDH Forward	CAAGGCTGAGAACGGGAAG
GAPDH Reverse	TGAAGACGCCAGTGGACTC
Divergent GAPDH Forward	GAAGGTGAAGGTCGAGTC
Divergent GAPDH Reverse	GAAGATGGTGATGGGATTTC
hsa-miR-636 Forward	ACACTCCAGCTGGGTGTGCTTGCTCGTCCCG
hsa-miR-1250-5p Forward	ACACTCCAGCTGGGACGGTGCTGGATGTG
hsa-miR-578 Forward	ACACTCCAGCTGGGCTTCTTGTGCTCTAG
miRNA reverse primer	TGGTGTCGTGGAGTCG
U6 Forward	CTCGCTTCGGCAGCACA
U6 Reverse	AACGCTTCACGAATTTGCGT
18S Forward	GGAGTATGGTTGCAAAGCTGA
18S Reverse	TCCTGCTTTGGGGTTCGATT
**oligonucleotides**	
Si-NC sense	UUCUCCGAACGUGUCACGUTT
Si-NC antisense	ACGUGACACGUUCGGAGAATT
CircPTPRA-si1 sense	CCCUCCUUCAGAUAAGCAUTT
CircPTPRA-si1 antisens	AUGCUUAUCUGAAGGAGGGTT
CircPTPRA-si2 sense	CUUCAGAUAAGCAUGGAUUTT
CircPTPRA-si2 antisens	AAUCCAUGCUUAUCUGAAGTT
mimic-NC sense	UUCUCCGAACGUGUCACGUTT
mimic-NC antisense	ACGUGACACGUUCGGAGAATT
miR-636 mimic sense	UGUGCUUGCUCGUCCCGCCCGCA
miR-636 mimic antisense	UGCGGGCGGGACGAGCAAGCACA
Inhibitor-NC	CAGUACUUUUGUGUAGUACAA
miR-636 inhibitor	UGCGGGCGGGACGAGCAAGCACA
KLF9-si1 sense	GCAUGAUCAAGCGAUCGAATT
KLF9-si1 antisense	UUCGAUCGCUUGAUCAUGCTT
KLF9-si2 sense	CUCUCUUGAAUCACGUUAATT
KLF9-si2 antisense	UUAACGUGAUUCAAGAGAGTT
**Probes**	
CircPTPRA-5’Cy3	AACCAGGAATCCATGCTTATCTGAAGGAGGGAAAGTTTCT
miR-636-5’Cy5	TGCGGGCGGGACGAGCAAGCACA
U6-5’Cy3	TTTGCGTGTCATCCTTGCG
18S-5’Cy3	CTTCCTTGGATGTGGTAGCCGTTTC
CircPTPRA-5’biotin	AACCAGGAATCCATGCTTATCTGAAGGAGGGAAAGTTTCT
miR-636-5’biotin	UGUGCUUGCUCGUCCCGCCCGCA
miR-636 mutant-5’biotin	UCACGAAGCUCGUCCCGCCCGCA
